# Impacts of cellulase deactivation at the moving air–liquid interface on cellulose conversions at low enzyme loadings

**DOI:** 10.1186/s13068-019-1439-2

**Published:** 2019-04-23

**Authors:** Samarthya Bhagia, Charles E. Wyman, Rajeev Kumar

**Affiliations:** 1 Department of Chemical and Environmental Engineering, Bourns College of Engineering, University of California Riverside, 900 University Ave, Riverside, CA 92521 USA; 2 Center for Environmental Research and Technology (CE-CERT), Bourns College of Engineering, University of California Riverside, 1084 Columbia Avenue, Riverside, CA 92507 USA; 3 BioEnergy Science Center (BESC), Oak Ridge National Laboratory, PO Box 2008 MS6341, Oak Ridge, TN 37831 USA; 4 Center for Bioenergy Innovation (CBI), Oak Ridge National Laboratory (ORNL), Oak Ridge, TN USA

**Keywords:** Cellulose, Cellulase, Deactivation, Hydrolysis, Air–liquid interface, Gas–liquid interface

## Abstract

**Background:**

We recently confirmed that the deactivation of *T. reesei* cellulases at the air–liquid interface reduces microcrystalline cellulose conversion at low enzyme loadings in shaken flasks. It is one of the main causes for lowering of cellulose conversions at low enzyme loadings. However, supplementing cellulases with small quantities of surface-active additives in shaken flasks can increase cellulose conversions at low enzyme loadings. It was also shown that cellulose conversions at low enzyme loadings can be increased in unshaken flasks if the reactions are carried for a longer time. This study further explores these recent findings to better understand the impact of air–liquid interfacial phenomena on enzymatic hydrolysis of cellulose contained in Avicel, Sigmacell, α-cellulose, cotton linters, and filter paper. The impacts of solids and enzyme loadings, supplementation with nonionic surfactant Tween 20 and xylanases, and application of different types of mixing and reactor designs on cellulose hydrolysis were also evaluated.

**Results:**

Avicel cellulose conversions at high solid loading were more than doubled by minimizing loss of cellulases to the air–liquid interface. Maximum cellulose conversions were high for surface-active supplemented shaken flasks or unshaken flasks because of low cellulase deactivation at the air–liquid interface. The nonionic surfactant Tween 20 was unable to completely prevent cellulase deactivation in shaken flasks and only reduced cellulose conversions at unreasonably high concentrations.

**Conclusions:**

High dynamic interfacial areas created through baffles in reactor vessels, low volumes in high-capacity vessels, or high shaking speeds severely limited cellulose conversions at low enzyme loadings. Precipitation of cellulases due to aggregation at the air–liquid interface caused their continuous deactivation in shaken flasks and severely limited solubilization of cellulose.

**Electronic supplementary material:**

The online version of this article (10.1186/s13068-019-1439-2) contains supplementary material, which is available to authorized users.

## Background

Conversion of crystalline cellulose into cellobiose by cellulases and conversion of cellobiose into glucose by β-glucosidases are important for enzymatic saccharification to achieve near theoretical glucose yields at mild conditions [1, 2]. However, since cellulases lose activity over reaction time [3], better understanding of cellulase deactivation can help devise strategies for making this process more economical. Surface tension forces were reported to play a significant role in causing cellulase deactivation by Reese and co-workers [3–5] and Jones and Lee [6] in the 1980s. But other studies (including Reese ad Ryu [7]) proposed mechanisms such as shear stress [8], changes in exoglucanase–endoglucanase synergy [9, 10], immobilization of enzyme on substrate [11], and thermal deactivation [12] for surfactant or shaking effects. Studies of enzymatic hydrolysis of cellulosic substrates were generally carried out for short reaction times, from a few hours to 5 days [2, 13]. However, at low enzyme to substrate ratios, glucose yields from pure and low-lignin cellulosic substrates continue to rise significantly beyond 5 days [14]. Overall, the result is that the cause of cellulase deactivation was still elusive.

In our recent work [14], it was shown that deactivation of fungal cellulase from a *T. reesei* hypercellulolytic mutant at the air–liquid interface was the main cause of lower cellulose conversion of Avicel PH-101 and low-lignin lignocellulosic biomass at 1% glucan substrate loadings for application of low enzyme to substrate ratios (5 mg, equivalent to ~ 2.5 FPU [15], of DuPont Accellerase^®^ 1500 per g glucan). This work was designed to understand why shaking had a negative effect on cellulose conversion and how additives improved cellulose conversion [14]. For better understanding of cellulase deactivation, the reactions were monitored till there was no appreciable increase in product yield as negative effect of shaking can appear late in the reaction: in our earlier work, 17 days was enough reaction time to get close to maximum possible glucose yields. While Avicel cellulose conversion plateaued at ~ 60% with shaking, 90–95% conversion was achieved by lowering enzyme deactivation at the interface through either not shaking the flasks or shaking flasks to which a small amount of a surface-active additive (5 mg Tween 20 or bovine serum albumin per g glucan) was added, over 17 days of reaction. This study revealed that deactivation of cellulase at the air–liquid interface was caused by its partial unfolding of cellulase to expose its hydrophobic regions to air to increase entropy and thus reduce free energy [14].

The work reported here employed new experiments to further clarify the effect of the air–liquid interface on cellulose conversions at low enzyme loadings, along with a more thorough investigation of mechanism responsible for reducing enzyme effectiveness. One part investigated the effects of shaking or surfactant addition on hydrolysis of cellulosic substrates other than Avicel. To confirm that deactivation was not specific to a particular commercial enzyme preparation, Novozymes Cellic^®^ CTec2^®^ was used for these experiments following the procedures previously carried out with Dupont’s Accellerase^®^ 1500 cellulase. In addition, new experiments were introduced to determine the effects of interfacial area of static flasks, surfactant loadings, enzyme loadings, xylanase supplementation, air–liquid interface in the reaction flasks, shaking mode, and solids loading on hydrolysis. Xylanases were applied to observe how yields for shaken flasks compared with those from unshaken flasks and surfactant-supplemented shaken flasks. The intent of including long reaction times was to determine maximum cellulose conversions to improve the understanding of cellulase behavior, but such long reaction time are not likely to be attractive for a commercial-scale saccharification process.

## Results

Throughout this paper, the term “enzyme” means Accellerase^®^ 1500, except if noted to be Cellic^®^ CTec2, and the term “Tween” means Tween^®^ 20 nonionic surfactant. All protein or surfactant loadings were based on mg per g glucan in cellulosic substrate and are referred as “mg enzyme” or “mg Tween.” Based on the experimental errors, only changes beyond 2–3% are considered significant. Experiments for comparison of Accellerase 1500 with Cellic CTec2 on Avicel cellulose conversions at low enzyme loading (Additional file 1: Fig. S1), effects of changing interfacial area and surfactant supplementation in unshaken flasks on Avicel cellulose conversions (Additional file 1: Fig. S2) and effects of surfactant supplementation and shaking on enzymatic conversions of beechwood xylan with xylanase (Additional file 1: Fig. S3) are available in Additional file 1. The Additional file 2 contains cellulose conversion data for all figures in this paper.

### Effects of surfactant supplementation and shaking on enzymatic conversions of various model cellulosic substrates at low enzyme loading

**Fig. 1:**
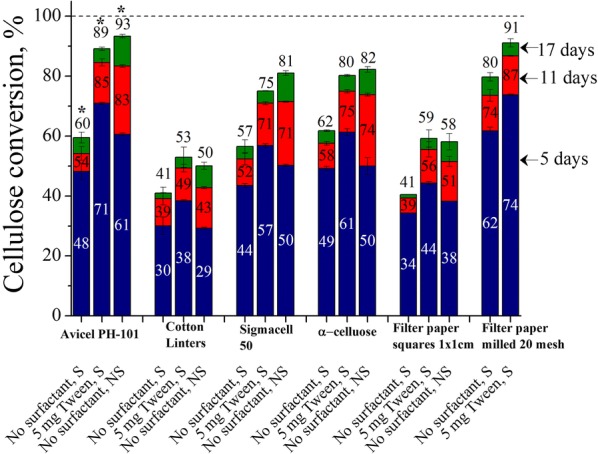
Effects of surfactant supplementation and shaking on conversions of cellulosic substrates at low enzyme loading. Cellulose conversions are reported after 5, 11, and 17 days of enzymatic hydrolysis of model cellulosic substrates at 1% glucan loadings using 5 mg cellulase protein (Accellerase^®^ 1500) per g glucan in shaken flasks, 5 mg Tween 20 per g glucan-supplemented shaken flasks, and unshaken flasks, all with a 50 mL reaction volume in 125 mL Erlenmeyer flasks. *S* shaking, *NS* no shaking. Data for Avicel conversions are from Bhagia et al. [14]

To determine how shaking and supplementation with surface-active additives affect cellulosic substrates other than Avicel, enzymatic conversions of popular commercially available celluloses were monitored over 17 days of reaction (Fig. 1). Digestibility of the celluloses followed the order: cotton linters < Whatman No. 1 filter paper squares 1 cm^2^ < Sigmacell Type 50 < Avicel PH-101 < α-cellulose < Whatman No. 1 filter paper milled through 20-mesh (< 0.850 mm) screen. Cellulose conversions for all substrates with 5 mg enzyme were improved by either supplementation with 5 mg Tween in shaken flasks or stopping shaking. However, the absolute increases in the maximum cellulose conversion by supplementing with Tween (i.e., the yield from surfactant-supplemented shaken flasks minus the yield from shaken flasks without surfactant) were different: 29% for Avicel PH-101, 12% for cotton linters, 18% for Sigmacell Type 50, 18% for α-cellulose, 18% for filter paper squares, and 11% for milled filter paper. Thus, the greatest improvement was for Avicel. Milling of the same material, i.e., filter paper, enhanced the digestibility by increasing surface area and reduced the benefit of surfactant supplementation. Moreover, conversions for Avicel and Sigmacell celluloses in unshaken flasks were higher after 17 days than results for shaken flasks supplemented with 5 mg Tween.

### Effect of baffles in shaken Erlenmeyer flasks on Avicel cellulose conversions at low enzyme loading

**Fig. 2:**
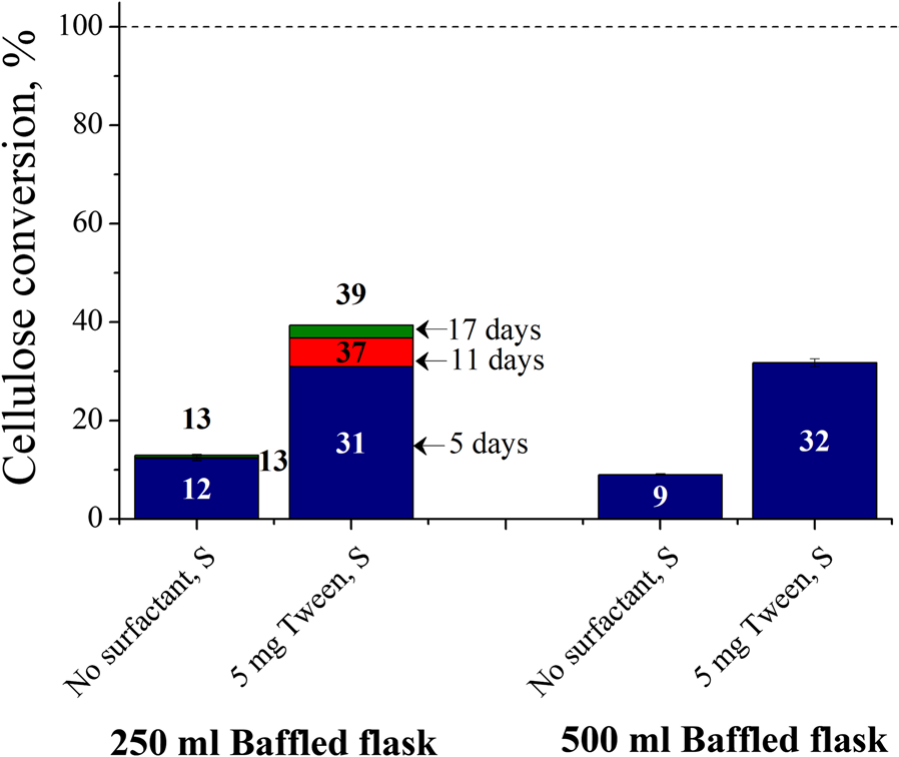
Effect of baffles in shaken Erlenmeyer flasks on Avicel cellulose conversions at low enzyme loading. Cellulose conversions are reported after 5, 11, and 17 days of enzymatic hydrolysis of Avicel cellulose at 1% glucan loading using 5 mg cellulase protein (Accellerase^®^ 1500) per g glucan in shaken flasks and 5 mg Tween 20 per g glucan-supplemented shaken flasks, at 50 mL reaction volume in 250 and 500 mL deep-baffled flasks. *S* shaking

Reactions were carried out in shaken baffled Erlenmeyer flasks to determine how Avicel cellulose hydrolysis is affected when the air–liquid interfacial area is greatly increased. Figure 2 shows that the maximum Avicel cellulose conversion was 13% without surfactant in 250 mL shaken baffled flasks. Conversions were even lower for reaction with the same 50 mL reaction contents in 500 mL baffled shaken flasks. While the addition of 5 mg Tween roughly tripled the cellulose conversions for both flasks after 17 days. However, this surfactant amount was not enough to result in high conversions at low 5 mg enzyme loading as seen in conventional flasks (non-baffled; Fig. 1).

### Effects of surfactant supplementation and shaking on cellulose conversions of Avicel vs. cotton linters at a high enzyme loading

**Fig. 3:**
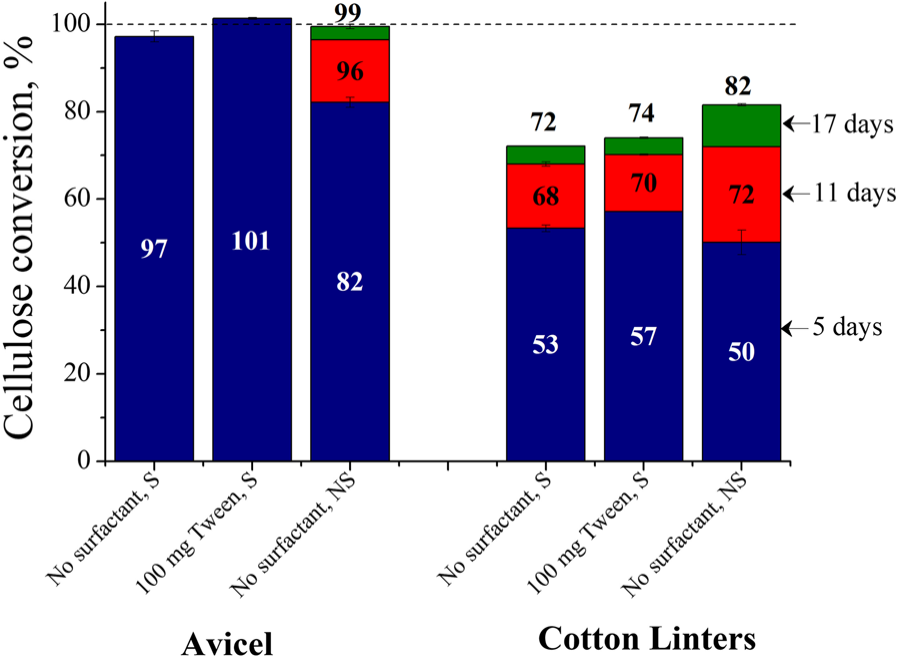
Cellulose conversions of Avicel vs. cotton linters at a high enzyme loading. Cellulose conversions are reported after 5, 11, and 17 days of enzymatic hydrolysis of Avicel and cotton linters at 1% glucan loading using 30 mg cellulase protein (Accellerase^®^ 1500) per g glucan in shaken flasks, 100 mg Tween 20 per g glucan-supplemented shaken flasks and unshaken flasks, at 50 mL reaction volume in 125 mL Erlenmeyer flasks. *S* shaking, *NS* no shaking

As discussed earlier [14], the ratio of enzyme deactivated at the interface to active enzyme was low at a high enzyme loading of 30 mg. As a result, supplementation with surface-active additive in shaken flasks or saccharification without shaking had little impact on maximum Avicel cellulose conversion. This result can be explained by the high amount of active enzyme rapidly solubilizing Avicel cellulose almost completely before much enzyme activity can be lost. Since cotton linters are more recalcitrant, interfacial deactivation of cellulase may have an impact even at high enzyme loadings. Therefore, experiments were carried out at 30 mg enzyme loading to compare cellulose conversions of Avicel and cotton linters through surfactant-supplementation and shaking. Figure 3 shows these trends as with 30 mg enzyme Avicel cellulose was solubilized rapidly and completely. As with Avicel, conversion of cotton linters had negligible increase in conversion when 30 mg enzyme was supplemented with 100 mg Tween. But conversions in static flasks were only 2–4% points higher than shaken flasks after 11 days. The largest benefit of not shaking the flasks came between 11 and 17 days, as cotton linters cellulose conversion in unshaken flasks was higher by 8–10% than for shaken flasks after 17 days even at this high enzyme loading. For both Avicel and cotton linters, while Fig. 3 shows that their 5 days of cellulose conversions were lower in unshaken flasks than shaken flasks at high enzyme loading, Fig. 1 showed that their 5 days of cellulose conversions were higher or similar in unshaken flasks than shaken flasks at low enzyme loadings.

### Comparison of Avicel cellulose conversions for shaking vs. stirring at low and high enzyme loadings

**Fig. 4:**
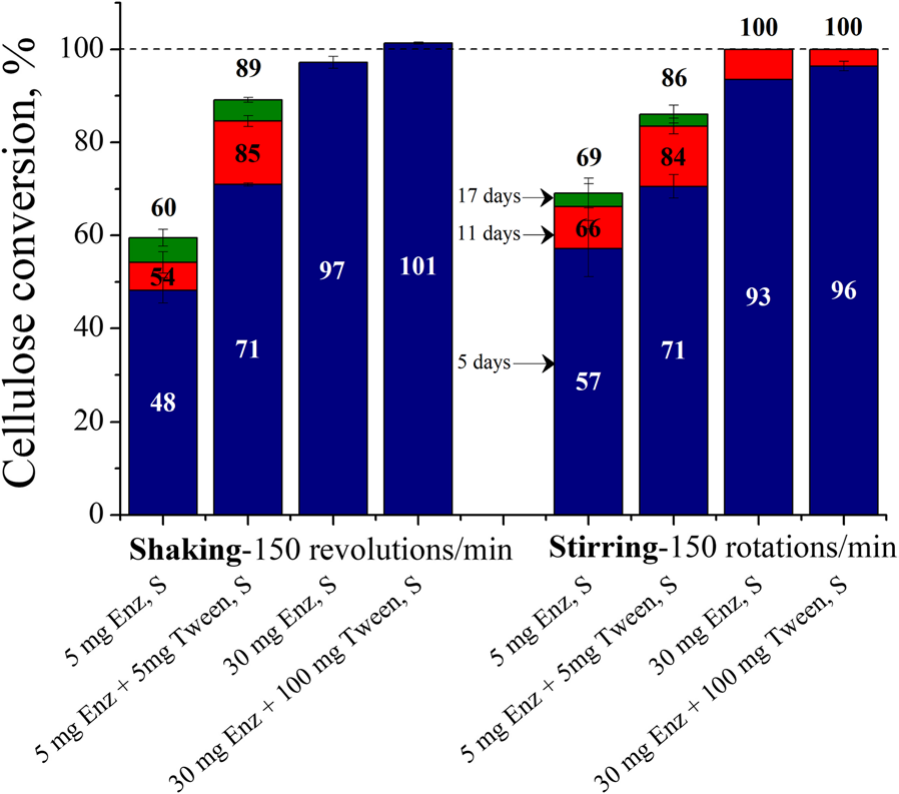
Comparison of Avicel cellulose conversions for shaking vs. stirring at low and high enzyme loadings. Cellulose conversions are reported after 5, 11, and 17 days of enzymatic hydrolysis of Avicel PH-101 cellulose at 1% glucan loading using 5 or 30 mg cellulase protein (Accellerase^®^ 1500) per g glucan and when supplemented with 5 or 100 mg Tween 20 per g glucan, at 50 mL reaction volume, in 125 mL orbitally shaken and magnetically stirred Erlenmeyer flasks at 150 rpm. *S* shaking

Orbital shakers or magnetic stirrers are generally used to mix laboratory-scale enzymatic hydrolysis flasks. While used interchangeably and generally considered equivalent, differences in dynamic interfacial area can affect the ratio of inactive to active enzyme. Figure 4 shows that in the absence of surfactant, Avicel cellulose conversions with 5 mg enzyme were lower at any given time with shaking than stirring, with stirring increasing yields by 9 percentage points at the end of reaction. However, supplementation at low enzyme loadings with 5 mg Tween or enzymatic hydrolysis at a higher 30 mg enzyme loading irrespective of surfactant addition virtually eliminated differences in cellulose conversions between shaking and stirring.

### Effects of Tween 20 supplementation and shaking on Avicel cellulose conversions using 10 mg of cellulase or 5 mg cellulase + 5 mg xylanase

**Fig. 5:**
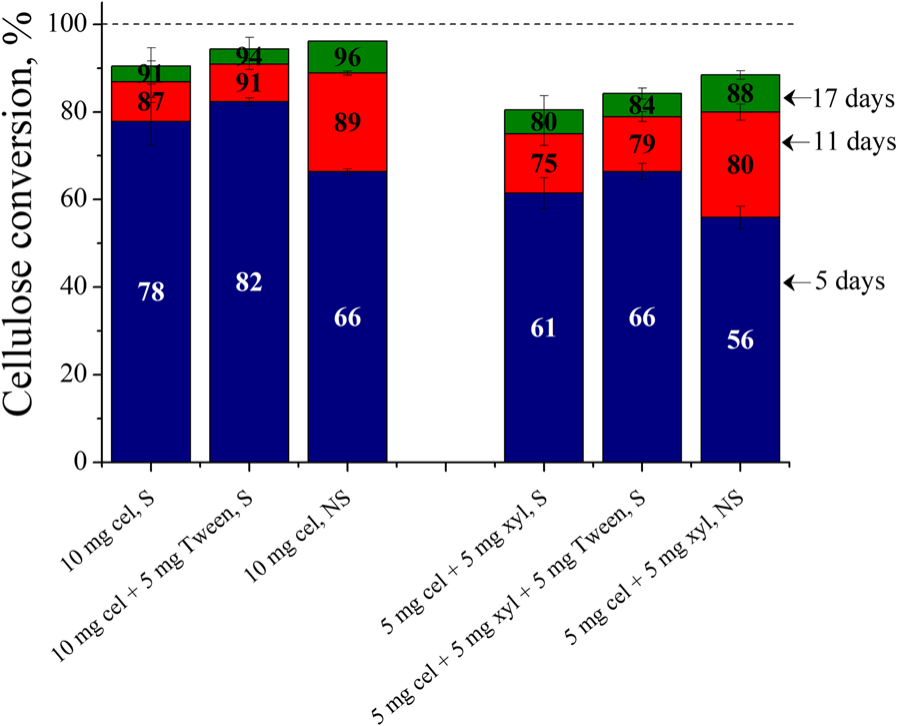
Avicel cellulose conversions using 10 mg cellulase or 5 mg cellulase + 5 mg xylanase. Cellulose conversions are reported after 5, 11 and 17 days of enzymatic hydrolysis of Avicel cellulose at 1% glucan loading using 10 mg cellulase protein (Accellerase^®^ 1500) or 5 mg cellulase + 5 mg xylanase protein per g glucan in shaken flasks, 5 mg Tween 20 per g glucan-supplemented shaken flasks and unshaken flasks, at 50 mL reaction volume in 125 mL Erlenmeyer flasks. *Cel* cellulase, *xyl* xylanase, *S* shaking, *NS* no shaking

Next, experiments were conducted to determine the approximate enzyme loading at which air–liquid interfacial deactivation has a limited influence on enzymatic hydrolysis of the well-studied microcrystalline cellulose substrate, Avicel. Figure 5 shows that when the enzyme loading was doubled from 5 to 10 mg, supplementation with 5 mg Tween increased Avicel cellulose conversions by only 3–4% at any reaction time. While unshaken flasks had lower reaction rates, all flasks with 10 mg enzyme loading reached greater than 90% conversion. When the reaction was allowed to go to completion, unshaken or surfactant-supplemented shaken flasks had 3–5% higher conversions, indicating a diminished effect of air–liquid interfacial deactivation of cellulase at this enzyme loading.

In another set of experiments, the effect of supplementing 5 mg of Accellerase^®^ 1500 cellulolytic enzyme with 5 mg of Accellerase^®^ XY xylanolytic enzyme was studied. Although Accellerase 1500 primarily contains cellulase and β-glucosidase activities, Accellerase^®^ XY has mostly xylanases and β-xylosidase activities [16]. As shown in Fig. 5, Avicel cellulose conversions with 5 mg cellulolytic enzyme + 5 mg xylanolytic enzyme were lower than those with 10 mg of just Accellerase^®^ 1500. Thus, while Fig. 1 shows that 17-day conversions with 5 mg Accellerase^®^ 1500 alone were restricted to 60% in shaken flasks, Fig. 5 shows that supplementation with 5 mg Accellerase^®^ XY increased 17-day conversion to 80%. However, when supplemented with 5 mg Accellerase^®^ XY (Fig. 5), Avicel cellulose conversions were lower by 3–6% with both 5 mg enzyme in unshaken flasks and 5 mg enzyme + 5 mg Tween in shaken flasks at any of the measured reaction times compared to the same conditions without 5 mg Accellerase^®^ XY (Fig. 1).

### Effects of reactor design and reaction volume on Avicel cellulose conversions at low enzyme loading

**Fig. 6:**
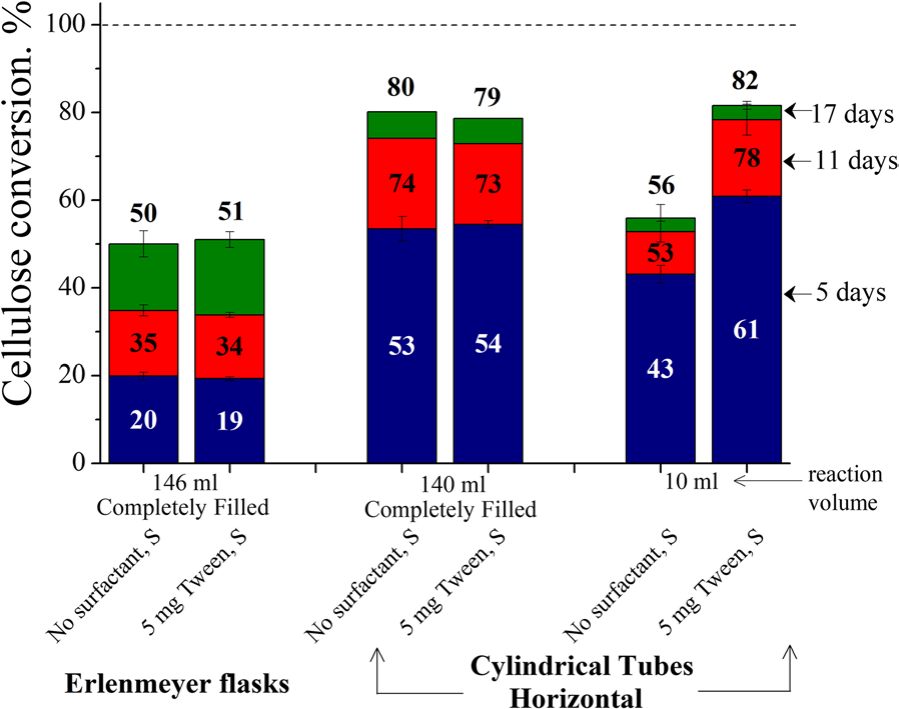
Effects of reactor design and reaction volume on Avicel cellulose conversions at low enzyme loading. Cellulose conversions are reported after 5, 11 and 17 days of enzymatic hydrolysis of Avicel cellulose at 1% glucan loading using 5 mg cellulase protein (Accellerase^®^ 1500) per g glucan and when supplemented with 5 mg Tween 20 per g glucan at three reactor styles [1] completely filled (146 mL r. vol.) 125-mL Erlenmeyer shaken flasks [2] completely filled (140 mL r. vol.) horizontal shaken tubes [3] 10 mL r. vol. in horizontal shaken tubes. *S* shaking

While surfactant addition or stopping of shaking can reduce air–liquid interfacial enzyme deactivation, filling an Erlenmeyer flask to the brim can greatly limit exposure of liquid to air by coupling a significant reduction in interfacial area and minimizing liquid movement even if shaken. Thus, the reaction volume was increased to 146 mL in 125 mL-rated Erlenmeyer flasks to completely fill the glass reactor at the same 1% glucan solid loading and same enzyme to substrate ratio as before. Results from these “completely filled” Erlenmeyer flask experiments were surprising, as instead of the expected increase in Avicel conversions due to near complete removal of interfacial enzyme deactivation, Fig. 6 shows how the conversions plummeted with Avicel cellulose conversions merely 20% after 5 days and reaching about 50% after 17 days. In addition, Tween addition had no impact on conversion. It was observed that there was absolutely no movement of solids in the heterogenous reaction medium when flasks were shaken at 150 rpm in orbital shaker i.e., shaken flasks mimicked unshaken flasks. To explain this surprising result, it was hypothesized that the dramatic drop in conversions was due to localized build-up of glucose causing end-product inhibition of cellulase. To test this hypothesis, 140 mL pressure tubes, similar to the ones recommended for the NREL biomass compositional analysis procedure and denoted here as “cylindrical tubes,” were used, as the Teflon screw-caps provided a leak-proof and inert seal when tubes were laid flat in the shaker. To these tubes were added 10 mL and 140 mL (complete filling) of reaction volume at the same 1% glucan loading as before. Nonetheless, an air bubble that moved one end to the other during shaking was always present even when the glass tubes were completely filled. However, unlike completely filled 125 mL Erlenmeyer flasks, in which the solids were stagnant when shaken, the Avicel solids reciprocated slightly along the tube wall in “completely filled” horizontally aligned tubes. Figure 6 shows that the final conversions were 30% points higher, supporting the hypothesis that localization of glucose in the vicinity of cellulose solids caused the drop in conversions for “completely filled” 125 mL Erlenmeyer flasks. In this case as well, Tween addition had no effect on conversions. Reducing the reaction volume to 10 mL in these tubes, however, resulted in the distinct positive impact of supplementing surface-active additive reappearing. Reaction rates were higher in 10 mL Tween-supplemented shaken tubes than for 140 mL Tween-supplemented shaken tubes, but the final conversions for both were similar, indicating that mixing was only slightly inefficient in completely filled shaken tubes.

### Effects of shaking on Avicel cellulose conversions at high solids loading with a low enzyme loading

**Fig. 7:**
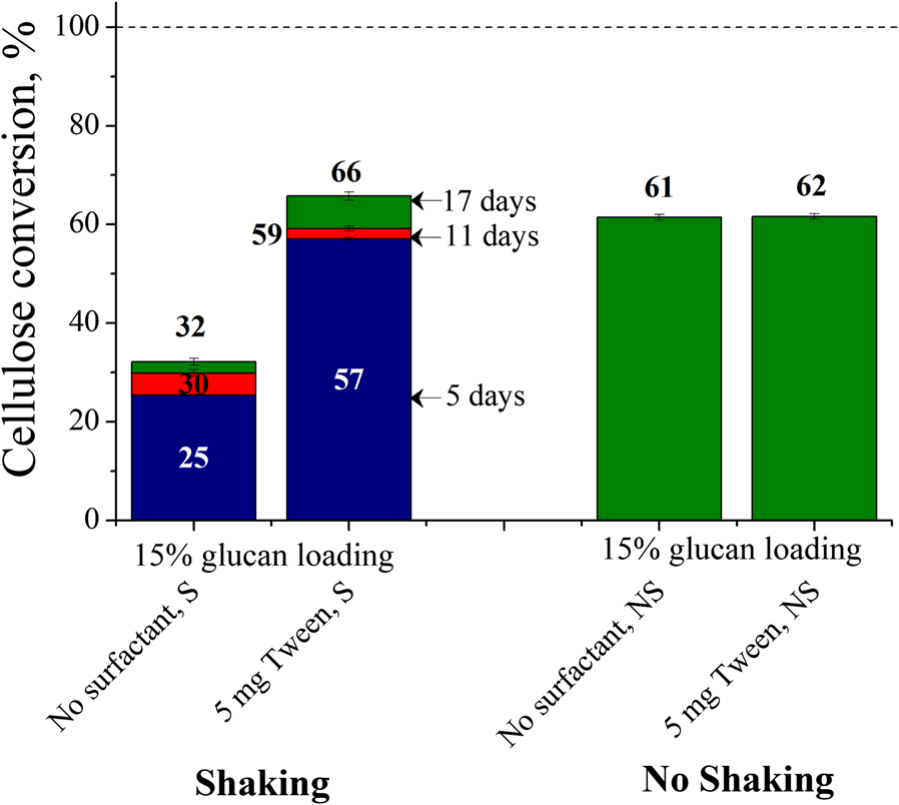
Effects of shaking on Avicel cellulose conversions at high solid loading and low enzyme loading. Cellulose conversions are reported after 5, 11, and 17 days of enzymatic hydrolysis of Avicel cellulose at 15% glucan loading using 5 mg cellulase protein (Accellerase^®^ 1500) per g glucan and supplemented with 5 mg Tween 20 per g glucan in shaken and unshaken flasks at 50 mL reaction volume in 125 mL Erlenmeyer flasks. For no shaking experiments, conversions were not measured after 5 and 11 days of reaction. *S* shaking, *NS* no shaking

Most of the interfacial phenomena experiments were carried out at a 1% glucan loading to minimize mass transfer limitations at higher substrate loadings. However, commercial-scale processes need to be carried out at high substrate loadings to keep processing costs as low as possible [17]. Figure 7 shows that for shaking without adding surfactant, the maximum cellulose conversion for a 15% Avicel glucan loading with 5 mg enzyme was 32%, about half that for a 1% Avicel glucan loading (Fig. 1). However, when 5 mg Tween per g glucan was added at a 15% glucan loading, cellulose conversion was roughly doubled (66%) in shaken flasks. This result is greater than the 60% maximum cellulose conversion achieved in shaken flasks for a 1% Avicel glucan loading with 5 mg enzyme but no surfactant. The most remarkable outcome of the high solid-loading experiments was for unshaken flasks as Fig. 7 shows that nearly 60% of Avicel cellulose was converted by 5 mg enzyme at a 1% glucan loading in shaken flasks or 15% glucan in unshaken flasks after 17 days. Similar to results for a l % glucan loading, Tween had little effect of conversions for the high 15% glucan loading in unshaken flasks.

### Effects of excessive Tween 20 surfactant concentrations on Avicel cellulose conversions at low enzyme loading

**Fig. 8:**
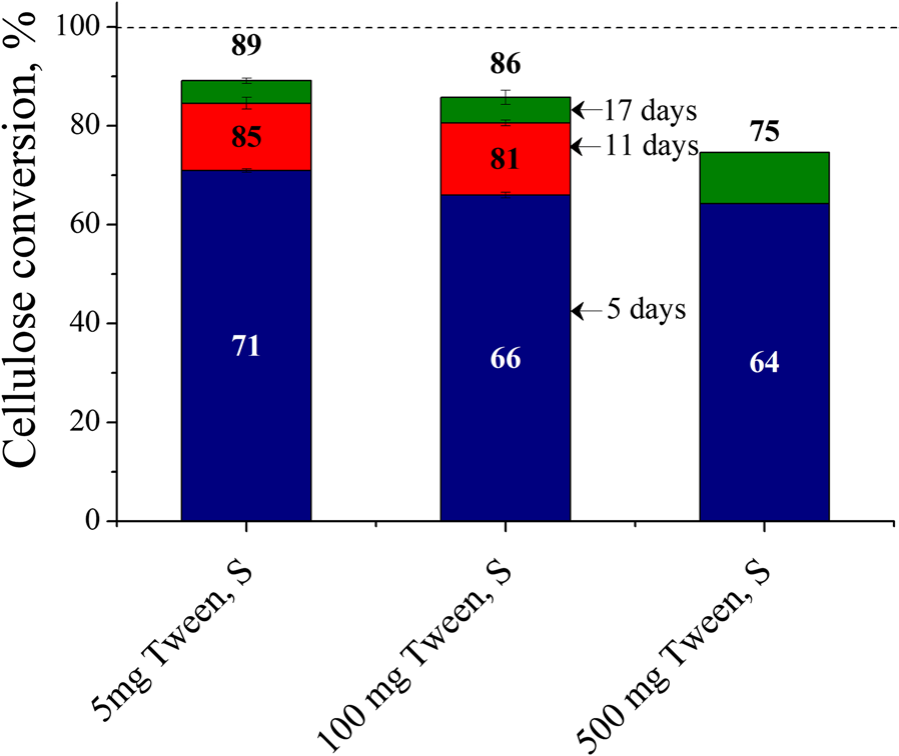
Effects of excessive Tween 20 surfactant concentrations on Avicel cellulose conversions at low enzyme loading. Cellulose conversions are reported after 5, 11 and 17 days of enzymatic hydrolysis of Avicel cellulose at 1% glucan loading using 5 mg cellulase protein (Accellerase^®^ 1500) per g glucan and when supplemented with 5, 100, or 500 mg Tween 20 per g glucan at 50 mL reaction volume in 125 mL Erlenmeyer shaken flasks. *S* shaking

Although it is known that a wide range of nonionic surfactant concentrations has little significant negative effect on enzymes [18], a recent paper reported that they may lower cellulose conversions at very high concentrations [19]. To verify this finding, Tween 20 concentrations were increased to identify the point at which it starts lowering cellulose conversions. Figure 8 shows that Avicel cellulose conversions dropped slightly by 3–5% points when Tween supplementation was increased by 20 times from 5 to 100 mg per g glucan (0.05 to 1 mg/mL) with 5 mg enzyme. Furthermore, increasing it even more by 100 times to 5 mg/mL dropped the final conversion by 14%.

## Discussion

### Effects of surfactant supplementation and shaking on enzymatic conversions of various model cellulosic substrates at low enzyme loading

Large improvements in cellulose conversion of several nearly pure celluloses either by supplementation with surface-active additive or stopping shaking showed the importance of reducing air–liquid interfacial deactivation of cellulase at low enzyme loadings (Fig. 1). Avicel^®^ and Sigmacell^®^ are commercial brands of microcrystalline cellulose that are recovered through alkaline removal of lignin from plant biomass followed by bleaching and then alkaline and acid hydrolysis [20]. Both Avicel PH-101 and Sigmacell Type 50 are 50 µm particles. Cotton linters, the leftovers (short fuzz) on cotton seeds after removal of cotton lint (staple cotton) in the ginning process, are a source of pure cellulose [21]. α-Celluloses are insoluble solids formed by treating pulp consecutively with 17.5% and 9.45% NaOH [22]. Whatman^®^ no. 1 paper is a commercial grade of filter paper made entirely from α-cellulose portion of cotton linters [23]. While all of these materials are largely Type I celluloses, differences in plant cell wall properties as well as chemical processing affect their crystalline content, degree of polymerization, enzyme accessibility, water swelling, crystallite dimensions, pore size distribution, and surface charge [24]. Differences in these properties affected the ability of cellulolytic enzymes to solubilize cellulose. Higher surface area of milled filter paper made it more digestible than filter paper squares. Lower improvement through addition of surfactant to shaken flasks in cellulose conversion of milled filter paper than filter paper squares was due to its high digestibility, as enough active enzymes were in solution to convert large portions of the cellulose despite enzyme deactivation at the air–liquid interface. Overall, for the same cellulosic material, either lowering substrate recalcitrance or increasing enzyme loading enough to realize high conversion reduces the impact of air–liquid interface enzyme deactivation on cellulose conversions. Past studies by Whitaker [25, 26] and Basu and Pal [27] in the 1950s concluded that the detrimental effect of shaking occurred only with insoluble substrates because conversions of carboxymethylcellulose (CMC) were not affected by shaking. On the other hand, Miller and Birzgalis [28] reported in 1961 that the high amounts of enzyme used in earlier studies masked the effect of shaking on CMC conversion, that is, the glucose concentration dropped from 1 mg/mL to slightly less than 0.1 mg/mL when the concentration of *Myrothecium verrucaria* QM 460 cellulase was reduced from 1 to 0.01 units over 168 h of reaction with CMC. In addition, 0.02% bovine serum albumin (BSA) had the same “protective effect” as 0.002% BSA. The last point from their research supports our findings and the hypothesis that only so much surface-active additive is sufficient to minimize surface tension [14].

### Mechanism of cellulase deactivation, and the effect of baffles in shaken Erlenmeyer flasks on Avicel cellulose conversions at low enzyme loading

There is a negative entropy change when water structures form around a hydrophobic moiety. Since air is hydrophobic, the hydrophobic moiety is driven toward the air to reduce contact with water. Therefore, proteins are attracted to the air–liquid interface because dehydration of hydrophobic regions causes a large increase in entropy [29]. The process may occur in four stages: bulk diffusion, adsorption at the interface, unfolding, and rearrangement [30]. Similar to mechanism proposed for the adsorption behavior of BSA, lysozyme, β-casein and β-lactoglobulin at the air–liquid interface [31], cellulases may first move from the bulk solution to the sublayer (layer below the air–liquid interface) and then overcome the energy barrier to adsorption at the interface. This sequence is likely followed by a change in conformation to make the hydrophobic regions protrude into the air phase that results in lowering of surface tension of water until an equilibrium is achieved. Surface tension is lowered because of less dissimilarity between air and water phases. Cellulases can undergo structural rearrangements and interactions with increasing interfacial concentration over time.

Reese and co-workers [4] showed that the Gibbs’ surface excess of cellulases from *T. reesei* QM9414 in 50 mM citrate buffer at 18 °C was 118 mg/m^2^ for protein concentration from 0.02 to 8 mg/mL. However, adsorption isotherms of BSA, lysozyme, and β-casein show that the surface concentrations are in the range of 2 to 5 mg/m^2^ for bulk protein concentrations between 0.001 and 0.1 mg/mL [32]. The much higher surface excess reported by Reese [4] likely results from the fact that unlike studies with pure proteins, cellulase stock solutions are a mixture of many proteins and only some of them are likely to have high surface activity. Moreover, surface excess would be lower in an actual cellulose hydrolysis experiment in unshaken flask because surface tension decreases with temperature. Based on 2–5 mg/m^2^ surface concentrations and operation at the conventional condition of a 50 mL reaction volume in a 125 mL Erlenmeyer flask, the ~ 20 cm^2^ approximate static interfacial area may deactivate only about 0.004–0.01 mg of 2.5 mg (5 mg protein per g glucan). This very low amount of deactivation can explain why the maximum cellulose conversions were highest in unshaken flasks. It is thus clear that the large drop in cellulose conversions caused by shaking must be due to continuous deactivation of cellulases over time. This outcome can result either by desorption of deactivated enzymes back into solution or their precipitation out of solution so that incoming active enzyme can adsorb into the air–liquid interfacial layer. But the energy barrier to desorption from the interface and back into bulk liquid phase has been calculated to be very high [33]. Therefore, after partial unfolding and rearrangement that favors closer packing, it is more likely that intermolecular hydrophobic interactions cause enzyme aggregation that leads to their precipitation when the aggregate size reaches beyond the solubility limit. This removal of enzyme from the interface maintains a concentration gradient for the bulk enzyme to keep adsorbing in the interfacial phase over time in shaken flasks. Thus, in shaken flasks, air–liquid interfacial deactivation of cellulase can be pictured as a five-step process: diffusion, adsorption, unfolding, rearrangement and aggregation, and precipitation. In shaken Erlenmeyer flasks, precipitation is evidenced by a prominent white ring on the reactor wall at the highest point liquid can reach (throw of liquid). Some of these precipitates fall back and are suspended in the liquid phase. Large dynamic interfacial area created through keeping reaction volumes low in large capacity Erlenmeyer shaken flasks [14], using baffles in shaken flasks (Fig. 2), or faster shaking, makes enzyme deactivation even more severe.

### Mechanism of surface-active additive, and the effects of surfactant supplementation and shaking on cellulose conversions of Avicel vs. cotton linters at a high enzyme loading

When surface-active additives are added to the reaction medium, they can occupy the interfacial sites and reduce adsorption of cellulase in the interfacial layer. Surfactants like Tween 20 have high surface activity, and studies with β-casein, β-lactoglobulin, and α-lactalbumin suggest that the displacement of protein at the interface by surfactants does not simply occur by desorption of individual protein molecules; rather, surfactants initially adsorb in defects in protein film and then grow in size to eventually collapse the protein network [34]. Solutions of BSA produce significant foam, as it quickly adsorbs to the air–liquid interface and changes conformation. Cellulase solutions at the same concentration do not produce any visible foam. This higher surface activity of BSA can displace cellulase from the air–liquid interface and result in trends in cellulose conversion that are strikingly similar to those for the non-ionic surfactant Tween 20. A minimum in surface tension of water is achieved at the critical micellar concentration (CMC) of Tween 20 in water: 1.95 × 10^−5^ M at 5 °C and 1.08 × 10^−5^ M at 30 °C. Addition of 0.1 M NaCl increases the CMC of Tween 20 in water to 2.88 × 10^−5^ M at 30 °C [35]. Based on these observations, the CMC of Tween 20 in enzymatic hydrolysis solution containing 0.05 M sodium citrate buffer and 0.02% sodium azide may be around 1 to 4 × 10^−5^ M. Addition of 5 mg Tween 20 per g glucan corresponds to 4.07 × 10^−5^ M (0.05 mg/mL), enough surfactant to cover most of the air–liquid interface. But even then, maximum cellulose conversions were always higher in unshaken flasks than surfactant-supplemented shaken flasks. This outcome indicates that a slightly higher amount of cellulases still adsorbed at the interface in surfactant-supplemented shaken flasks compared to unshaken flasks and that surfactants were unable to completely prevent cellulase deactivation in shaken flasks. The adsorption behavior of a binary solution of surfactant and cellulase in a moving interface is unknown but shaking could spread the interfacial film on reactor walls to greatly increase surface area, exposing a small amount of enzyme to the gas phase. This mechanism could explain why enzymatic hydrolysis of cotton linters even at high enzyme loadings significantly increased glucose yields in unshaken flasks very late in the reaction. Otherwise, there should have been no difference in cotton cellulose conversions during 11 and 17 days between surfactant-supplemented shaken flasks (100 mg Tween 20) and unshaken flasks at the high 30 mg enzyme loading (Fig. 3).

5 days of cellulose conversions of Avicel and cotton linters were lower in unshaken flasks than shaken flasks at high enzyme loading (Fig. 3) but 5 days of cellulose conversions of Avicel or cotton linters were higher or similar in unshaken flasks than shaken flasks at low enzyme loading (Fig. 1). This was because at high enzyme loading, the ratio of enzyme inactivated at the interface to active enzyme was so low that it had an insignificant effect on 5 days of cellulose conversions in shaken flasks, but lack of mixing made the reaction rates lower in unshaken flasks. However, at low enzyme loading, the ratio of enzyme inactivated at the interface to active enzyme was higher and played a significant role in lowering of cellulose conversions in shaken flasks that despite lack of mixing, higher active enzyme concentrations made cellulose conversions higher or similar in unshaken flasks than shaken flasks.

### Comparison of Avicel cellulose conversions for shaking vs. stirring at low and high enzyme loadings

Cellulose hydrolysis by cellulases at low enzyme loadings in the laboratory should preferably be carried out through stirring rather than shaking (at the same speed), as the dynamic air–liquid interfacial area is higher for the latter type of mixing due to high liquid film formation on walls of shaken flasks (Fig. 4). Due to more active enzyme, reaction rates and maximum cellulose conversions should thus be significantly higher when mixing is done by stirring for low enzyme loading experiments. Otherwise, addition of a small amount of surface-active additive could make differences in type of mixing disappear. Brethauer et al. [36] in 2011 reported that stirring of 1% Avicel glucan solution in the absence of enzyme (pre-stirring) for 25 g reaction mass in 125 mL Erlenmeyer flask using ~ 0.79″ magnetic stir bars at 500 rpm for 24 h at RT slightly reduced crystallinity index from 53 to 48%. However, any drop in crystallinity index of Avicel due to attrition action of stirring was not the cause of higher cellulose conversion in stirred flasks as otherwise 5 mg Tween-supplemented stirred flasks would have resulted in higher cellulose conversion than 5 mg Tween-supplemented shaken flasks (Fig. 4).

### Effects of Tween 20 supplementation and shaking on Avicel cellulose conversions using 10 mg of cellulase or 5 mg cellulase + 5 mg xylanase

10 mg (or ~ 5 FPU) protein per gram glucan loading for 1% Avicel cellulose loading in normal operating conditions with a 50 mL reaction volume in 125 mL Erlenmeyer flasks at 150 rpm is roughly the limit at which the enzyme deactivation had minimal impact on cellulose hydrolysis as there was sufficient active enzyme to convert greater than 90% Avicel cellulose (Fig. 5). However, these conditions are too specific, but still useful as Avicel conversions over time are often tracked as a positive control along with test substrates in enzymatic hydrolysis experiments to ensure consistency of cellulase stock solutions. It might be more affordable to convert Avicel cellulose using 5 mg enzyme + 5 mg surfactant than 10 mg enzyme in shaken flasks because Avicel cellulose conversions were only slightly lower after 5 reaction days in the former case and little differences beyond 5 days.

The 20% increase in Avicel cellulose conversion after 17 days as a result of supplementing 5 mg cellulase with 5 mg xylanase in shaken flasks shows that the latter also competes with cellulases for interfacial sites (Fig. 5). All proteins are amphiphilic and tend to migrate to an air–liquid interface. As discussed previously [14], many amphiphilic additives are capable of causing such an effect, but since their surface activities are different, their ability to lower cellulase adsorption at the air–liquid interface is different. The 10% lower increase in conversion achieved by cellulase with 5 mg xylanase compared to supplementing with 5 mg Tween (in this work) or 5 mg BSA [14] in shaken flasks shows that the latter were better than xylanases at lowering cellulase deactivation. However, when cellulase deactivation at the interface was a minimum, i.e., in unshaken or surfactant-supplement shaken flasks, the 3–6% drop in Avicel cellulose conversions with 5 mg xylanase supplementation was likely due to xylanase binding to cellulose to reduce cellulose sites available for cellulase binding. This possibility was inferred earlier by Qing and Wyman [37] for a similar outcome in which Avicel cellulose conversion dropped by 7% after 5 days of reaction when 16 mg xylanase (Multifect^®^ xylanase) was added to 16 mg cellulase (Spezyme CP cellulase). However, since enzyme loadings were higher in their study, the positive effect of xylanase on reducing cellulase deactivation at the interface was not observed.

### Effects of reactor design and reaction volume on Avicel cellulose conversions at low enzyme loading

The large drop in conversion when the Erlenmeyer flasks were completely filled with reaction volume to eliminate the air–liquid interface can be attributed to end-product inhibition [38, 39] as high local glucose concentration built-up near the solid substrate due to high height of liquid phase (Fig. 6). This mechanism was inferred from the observance of high Avicel cellulose conversions in shaken horizontal cylindrical tubes. Although these tubes had nearly the same reaction volume as completely filled shaken Erlenmeyer flasks, the wider reactor design and slight movement of solids promoted homogeneity of liberated glucose. In other words, while keeping everything else constant, a 50 mL reaction volume in 100 mL vertical cylindrical unmixed vessel will have lower local glucose inhibition than 100 mL reaction volume in the same vessel. From these results, it can be explained that in normal laboratory condition i.e., 1% substrate at 50 mL volume in 125 mL Erlenmeyer flasks, the lower reaction rate in unshaken flasks was caused by local product inhibition (Fig. 1). However, because not shaking the flasks also minimized cellulase deactivation, the maximum (17 days) cellulose conversions were higher for not shaking than for shaking the flasks at low enzyme loading. When enzymatic hydrolysis is carried out at higher enzyme loadings and short reaction times, cellulase deactivation at the air–liquid interface has insignificant impact on cellulose conversions, and unshaken flasks have lower cellulose conversion than shaken flasks, as is the situation for most studies [40].

In any case, these experiments show that completely filling the reactors can reduce the air–liquid interfacial area provided that the design and movement allow good mixing of soluble products for these heterogenous reactions. Moreover, since the surface-to-volume ratio of an industrial-scale stirred tank is significantly lower than laboratory-scale reactor, cellulase deactivation at the air–liquid interface would have lower impact at the industrial-scale. A study of cellulose hydrolysis at low enzyme loading in stirred tanks is needed to understand how such aspects as impeller diameter, type, and speed; baffles; liquid height; and operational scale could impact performance in a commercial reactor.

Cellulase deactivation at the air–liquid interface or lack thereof was reported by Reese and co-workers [3–5, 7] and Ooshima and co-workers [9, 10] in the 1980s (discussed previously [14]), Jones and Lee [6] in 1988 and Sawant, Joshi, and co-workers [8, 41] in 2000 and 2001. Jones and Lee used stainless steel balls in a stirred tank to combine ball milling with enzymatic hydrolysis. They found that relative enzyme activity was maintained when the tank was filled with liquid to provide a liquid-tight seal compared to 50% drop in relative activity at standard liquid height when other conditions were kept constant: 20 h of reaction at high 8 mg/mL enzyme concentration and under accelerated impeller speed of 700 rpm to increase deactivation. It is noteworthy that they concluded that newspaper hydrolysis was significantly enhanced in this attrition bioreactor and surface tension forces, not shear forces, were responsible for enzyme deactivation. About a decade later, Sawant, Joshi, and co-workers [8, 41] placed a horizontal baffle on top of the liquid in a stirred tank reactor and claimed that in absence of gas sparging, this method completely eliminated the air–liquid interface. They concluded that while air–liquid interfacial phenomenon was important when gas was sparged in the reactor, shear stress was the cause of the drop in cellulase activity. It seems that their method of placing a horizontal baffle on top of moving liquid did not minimize, let alone eliminate, the air–liquid interface that is on the order of a few nanometers, due to small air gaps between the liquid and solid disk under agitation in a stirred tank with vertical baffles. While shear in a stirred tank may lyse mammalian and microbial cell membranes, there is no explanation why proteins would be affected by shear at impeller speeds typical of stirred tanks or why cellulase deactivation increased from low to high impeller speeds without a threshold. It could be assumed that wall friction in narrow capillary tubing could cause protein unfolding at high flow rates, but it has been shown that this too requires extremely high shear rates (10^7^ s^−1^) [42]. A review article has discussed in detail how hydrodynamic shear alone rarely causes damage to proteins at conventional conditions [43].

### Effects of shaking on Avicel cellulose conversions at high solids loading with a low enzyme loading

Doubling of Avicel cellulose conversions at high solid substrate loading (15% glucan) at low cellulase loading in surfactant-supplemented shaken flasks or unshaken flasks (Fig. 7) may offer significant economic benefits if industrial operations are carried out at high solid loadings. Mixing is employed for improving heat transfer and dispersing the heterogenous medium evenly so that product inhibition such as that seen in “completely filled” experiments can be diminished. However, the effect of the air–liquid interface on deactivating cellulase was so severe in these high solid loading experiments that not shaking the reaction gave far better results than shaking the flasks. This outcome is valid only for long reactions times, as accumulation of end-products in the vicinity of solid substrate lowers reaction rates in unshaken reactors, as deduced from the extreme case of localized end-product inhibition that occurred in completely filled Erlenmeyer flasks. The maximum conversion in surfactant-supplemented shaken flasks was only 4–5% points better than unshaken flasks, but due to good mixing, close to 60% cellulose conversion could be realized in as little as 5 days. On the other hand, unlike for the laboratory-scale reactor, the surface-to-volume ratio of an industrial reactor is low, which demands mixing to reduce temperature gradients [44]. Therefore, as mixing is likely unavoidable at the commercial scale, it could be very beneficial to add a small quantity of a surface-active additive for high solids loading cellulose hydrolysis at low enzyme loadings. A complex nutrient source such as corn steep liquor is added in fermentation of biomass sugars into ethanol by yeast [45]. The complex nutrient sources may carry compounds that have high surface activity. Soy isolates contain lecithins and saponins that have been previously shown to improve sugar yields from enzymatic hydrolysis [14]. A small portion of the nutrient source for fermentation could be diverted to enzymatic saccharification vessel to fulfill the need for surface-active additive without increasing raw material cost.

The drop in the maximum Avicel cellulose conversion from ~ 90 to ~ 60% when glucan loading was increased from 1 to 15% can be attributed to a lower water concentration. In both cases, enzyme was loaded based on per unit mass of substrate, and for both cases, glucose (acting is inhibitor) to enzyme ratio was the same for the same level of cellulose conversion. However, both glucose and enzyme are at higher concentrations. Lesser amount of water is known to reduce cellulase adsorption on solid cellulose substrate surface [46, 47]. Cellulose conversions are, therefore, lower because only the adsorbed enzyme can form cellobiose. The exact cause of reduced adsorption at reduced water concentration is unclear but could be due altered water structuring or participation of water molecule in adsorption of binding domain of cellulase with cellulose through hydrogen bonding.

### Effects of excessive Tween 20 surfactant concentrations on Avicel cellulose conversions at low enzyme loading

Supplementation with Tween 20 at a concentration of about 5 mg Tween (0.05 mg/mL) was sufficient to realize high conversions of pure celluloses at low enzyme loadings. The drop in cellulose conversion that started to appear at 100 mg Tween (1 mg/mL) is evidence that even nonionic surfactants at high concentrations can have negative effects on enzymatic hydrolysis (Fig. 8). These results are consistent with another study [19] that showed a drop in microcrystalline cellulose and filter paper cellulose conversions at an enzyme loading of 5 FPU/g substrate with 1 and 5 g/L of Tween 20. However, such high surfactant loadings are unwarranted as CMC values are well below 0.1 mg/mL. The reason for lower cellulose conversions is not clear, but these results point to protein–surfactant interactions that need to be further explored.

### Endo–exo synergy and cellulase deactivation at the air–liquid interface

Our previous article [14] explained that other mechanisms such as thermal deactivation and changes to endoglucanase–exoglucanase (endo–exo) synergism were not responsible for such large changes in cellulose conversions as shaking should not influence either one. Thermal deactivation is marked by a change in protein conformation, and it does seem logical that fungal cellulases that are “thermostable” would be deactivated by heat at their optimum reaction temperature of 50 °C. Two exoglucanases, Cel7A and Cel6A (CBH I and CBH II), and two endoglucanases, Cel7B and Cel5A (EG I and EG II), account for the majority of cellulases secreted by *T. reesei* RUT-C30 and CL847, current cellulase overproducing strains [48]. Differential scanning calorimetry of purified cellulases in the 1992 study by Baker et al. [49] showed that heat capacities only start changing above 55 °C but then sharply above 60 °C due to the change in structure from native to denatured state. The melting temperatures (50% unfolded state) of Cel7A, Cel6A, and Cel7B are around 64 °C and Cel5A around 75 °C. This mechanism is further supported by both Eriksson et al. in 2002 [50] and Levine et al. in 2010 [51] who stated that neither thermal deactivation nor product inhibition could account for the drop in cellulose hydrolysis over time. But Ooshima, Sakata, and Harano still suggested that endo–exo synergism was affected by agitation in their 1985 paper [9] and that surfactant addition [10] also affected this synergy due to changes in adsorbed cellulase (endo–exo) composition in 1986. They were led to these conclusions because the 1985 paper [9] discounted air–liquid interface as a possible cause because reaction rates were not affected by changing reaction volume in shaken flasks. They could not have seen an effect of changing reaction volume because they used high enzyme loading at short reaction times that hid the effect of shaking on loss of cellulase activity [14]. While there is no explanation in these articles why agitation would affect endo–exo synergy, an argument could be made that since exoglucanases and endoglucanases are of different sizes, differences in their diffusion coefficients could affect the timescales of adsorption–desorption on the cellulose surface in unshaken flasks that somehow favors enzymatic hydrolysis, compared to shaken flasks where adsorption on cellulose is not diffusion-limited. Cel7A, Cel6A, Cel7B, and Cel5A have experimentally measured molecular weights (glycosylated) of 63, 55–59, 58, and 43–48 kDa, respectively. Assuming that these proteins are spherical and have an average partial specific volume of 0.73 cm^3^/g, diffusion coefficients of proteins can be calculated as *D* = 8.34 × 10^−8^ (*T*/*ηM*^1/3^) [52], where *D* is diffusion coefficient (cm^2^/s), *T* is temperature (K), *η* is viscosity (mPa s), and *M* is the molecular weight. Calculated diffusion coefficients for the first three enzymes in pure water at 50 °C fall between 1.24 and 1.30 × 10^−6^ cm^2^/s, a difference of only 4.4%, while that for Cel5A is 1.36–1.41 × 10^−6^ cm^2^/s, about 9.5–13.6% higher than for Cel7A. However, the first proposal that agitation shifts endo–exo synergy to favor cellulose conversions can neither explain why amphiphilic additives improve enzymatic hydrolysis of pure celluloses in shaken flasks nor why maximum cellulose conversions of additive-supplemented shaken flasks and unshaken flasks are strikingly close. The other proposal that a shift in endo–exo synergy by surfactant addition causes higher cellulose conversion cannot explain why surfactants have little effect in unshaken flasks. Air–liquid interfacial deactivation offers a clear and logical explanation that endo–exo synergy will be affected if deactivation of some cellulase activities at the air–liquid interface is greater than for others in the enzyme cocktail. Adsorption of all cellulases at the air–liquid interfaces is unlikely to be the same because differences in their physicochemical properties will affect their air–liquid surface activity. It has been long known that proteins have maximum adsorption at the air–liquid interface at their isoelectric point (pI) [53]. Due to lack of net charge at pI, there is no electric barrier to interfacial adsorption, and proteins may aggregate due to lack of repulsive forces [53]. Cel7A, Cel6A, Cel7B, and Cel5A have isoelectric points (pI) of 4.5–4.7, 5.0–5.2, 4.6–4.7, and 4.8–5.0, and GRAVY indices (grand average of hydropathy) [54] of − 4.33, − 0.18, − 0.37, and − 0.19 (larger value means protein is more hydrophobic), respectively. It is more likely that Cel6A and Cel5A had higher interfacial adsorption due to their higher hydrophobicity and isoelectric points that are closer to the reaction pH. Thus, higher air–liquid interfacial deactivation of some cellulases could affect endo–exo ratios in the bulk solution that in turn affect the endo–exo ratio available to adsorb on cellulose that could be perceived as a shift in endo–exo synergy.

Most articles that deal with the subject of loss activity of cellulase plot the relative activity over time. This process usually involves adding a known mass of enzyme and then removing aliquots from the enzymatic hydrolysis reaction at different times followed by applying the filter paper unit assay (FPU) [55]. It is now clear that as cellulases can precipitate out of solution in shaken flasks, protein assays and cellulase activity assays both need to be performed at all sampling times to correct for activity based on only the mass of soluble protein. It is important that the same reactor design, reactor materials of construction, reaction volumes, and shaking speeds be used in the laboratory for fair comparison of substrate recalcitrance in experimental enzymatic hydrolysis sets. Since the air–liquid interfacial phenomenon is fundamental to cellulase deactivation, a complete description of reaction conditions that includes reaction volume, vessel design, vessel material of construction, vessel capacity, shaking type, and shaking speed will facilitate better comparison and reproducibility among enzymatic hydrolysis studies.

Based on these findings of the deactivation of cellulases at the air–liquid interface on cellulose conversions, future experiments will measure cellulase activity, adsorption isotherm and surface tension to gain deeper insights into factors responsible for this phenomenon. Latest commercial cellulase preparations may contain lytic polysaccharide monooxygenases (LPMO) which need oxygen or hydrogen peroxide along with a reducing agent like ascorbic acid for efficient oxidative lysis of glycosidic bonds. Higher dissolved oxygen concentrations achieved through sparging with air or faster shaking may improve LPMO hydrolysis rates [56]. Therefore, cellulase deactivation at gas transfer conditions used for LPMO containing cellulases needs to be studied. Lastly, the additional file shows a video for some key experiments (Additional file 3). Of importance is the precipitation of cellulases after shaking that was carried out at a high enzyme loading of 100 mg (1 mg/mL) at 50 °C in 50 mM citrate buffer for 24 h by accelerating the deactivation through high shaking speed of 250 rpm.

## Conclusions

Cellulases are vulnerable to deactivation due to their aggregation and precipitation at the air–liquid interface in shaken flasks. Maximum cellulose conversions were high for surface-active supplemented shaken flasks or unshaken flasks because of low cellulase deactivation at the air–liquid interface. The nonionic surfactant Tween 20 was unable to completely prevent cellulase deactivation in shaken flasks and only reduced cellulose conversions at unreasonably high concentrations. Cellulose conversions were more than doubled by adding surface-active additives to cellulase in shaken flasks or not shaking the flasks for hydrolysis of high Avicel solid loading at low enzyme loading. High dynamic interfacial areas created through baffles in reactor vessels, low volumes in high-capacity vessels, or high shaking speeds severely limited cellulose conversions at low enzyme loadings. Thus, strategies to minimize the dynamic air–liquid interface while ensuring good mixing can provide a valuable approach to achieve high cellulose conversions at high reaction rates for low enzyme to substrate ratios.

## Methods

### Materials

Avicel^®^ PH-101 (~ 97% glucan content), Sigmacell Type 50 (~ 97% glucan content), cotton linters (100% glucan content), α-cellulose (~ 81 glucan content), Whatman^®^ qualitative filter paper grade 1 (~ 100% glucan content), and beechwood xylan (~ 70% xylan content) were purchased from Sigma-Aldrich Corp (St. Louis, MO). The glucan and xylan contents of the substrates were determined by following the standard two-step acid hydrolysis procedure (NREL/TP-510-42618) [57–59]. The Whatman filter papers were cut in approximately 1 × 1 cm^2^. For milled filter paper experiments, Whatman filter papers were knife-milled (Model 3383-L20 Wiley^®^ mill, Thomas Scientific, Swedesboro NJ) to pass through an ASTM 20 mesh screen (nominal sieve opening of 0.85 mm). Tween^®^ 20 (Acros Organics) and glassware were purchased from Fisher Scientific (Thermo Fisher Scientific Inc., Waltham, MA). Erlenmeyer flasks with rated-volumes of 25 mL (stoppered), 125 mL (screw cap), and 500 mL (screw cap), in addition to 250 mL (stoppered) and 500 mL (stoppered) rated extra-deep-baffled flasks (also known as trypsinizing flasks) were made of Pyrex^®^ borosilicate glass. For reactions in tubes, high pressure borosilicate glass tubes (Ace Glass, Inc., Vineland, NJ) were used. These tubes were ~ 8″ long below the neck, had a total volume of ~ 140 mL, and were sealed with Teflon screw plugs with O-rings to make them leak-proof. Accellerase^*®*^ 1500 (BCA protein content—82 mg/mL) and Accellerase^*®*^ XY (BCA protein content—51 mg/mL) were kind gifts from DuPont Industrial Biosciences (Palo Alto, CA). Novozymes Cellic^®^ CTec2 (BCA protein content—200 mg/mL) was a kind gift from Novozymes A/S (Franklinton, NC). The protein concentration of the stock solutions was determined by the bicinchoninic acid assay [60] using the microplate procedure of Pierce^*®*^ BCA Protein Kit (Thermo Fisher Scientific Inc., Waltham, MA).

### Enzymatic hydrolyses

All enzymatic hydrolysis runs were carried out according to the NREL standard procedure “Enzymatic Saccharification of Lignocellulosic Biomass” [61] with only the following modifications. All experiments were at 1 w/v% glucan loading of the cellulosic substrates or 1 w/v% xylan loading of beechwood xylan except high solids loading experiments that had 15 w/v% glucan loading of Avicel. The reaction was performed in 125 mL Erlenmeyer flasks with reaction volume of 50 mL in all experiments except those evaluating the effect of interfacial area in unshaken flasks for which 10 mL of reaction volume was used in 25, 125, or 500 mL unshaken Erlenmeyer flasks. The static interfacial areas measured with a ruler or Vernier calipers of 10 mL reaction volume for 25, 125, and 500 mL flasks were 7.9 cm^2^, 25.6 cm^2^, and 45.6 cm^2^, respectively. All enzymatic hydrolyses were carried out at 50 °C in 50 mM sodium citrate buffer at pH 5.0 with addition of 0.02% sodium azide broad-spectrum antibiotic using an orbital shaker (Multitron Standard, Infors^®^ HT Biotech, Laurel, MD) at 150 revolutions per minute for shaking experiments and 0 rpm for those without shaking, except those that compared shaking with stirring. The stirred reactions were in 50 mL reaction volumes in 125 mL Erlenmeyer flasks with 1.5″ long PTFE magnetic stirring bars at 150 rotations/min using a multi-point magnetic stirring plate (Thermo Scientific Poly 15 stirrer, Thermo Fisher Scientific Inc.) that was kept under a transparent acrylic heated water bath circulator at 50 °C. The 150 rpm for stirring or shaking was set using the magnetic stirring plate or orbital shaker’s speed settings. Stock enzyme solutions were diluted 20 times in 50 mM pH 5.0 citrate buffer in 100 mL borosilicate glass volumetric flasks for better accuracy in pipetting for reactions at low enzyme loadings. Due to their high foaming tendency, 1% stock solutions of Tween 20 were made by weighing 1 g of Tween 20 in a tared 125 mL conical flask followed by addition of milli-Q water to reach a total mass of 100 g for accuracy and reproducibility.

Accellerase^®^ 1500 and Tween 20 were loaded based on milligrams of protein or surfactant per gram glucan in the substrate. In all experiments including no shaking experiments, the reaction medium was shaken gently after enzyme addition. Tween 20 was added to Erlenmeyer flasks soon after the enzyme (co-addition). Loading of Accellerase^®^ 1500 was either 5 or 30 mg protein per gram glucan in cellulosic substrate. 5 mg or 100 mg of Tween 20 was also added based on grams of glucan in cellulosic substrates. Enzymatic hydrolysis at each condition was carried out in triplicate. Error bars in all figures represent sample standard deviation from the triplicates. Sampling was at 120, 264, and 408 h (5, 11, and 17 days) for all conditions by withdrawing 300 µL homogenous aliquots. However, for high solids loading (15%) unshaken flasks, homogenous aliquots were taken only at 408 h. For no shaking experiments, separate flasks were kept for each time point (120, 264, and 408 h) to not to disturb the reaction medium.

The aliquots were centrifuged at 15,000 rpm for 5 min in a fixed-angle centrifuge (Eppendorf^®^ Microcentrifuge Model 5424, Eppendorf North America, Hauppauge, NY). The supernatants were analyzed on a Waters^®^ e2695 Separations Module with detection on Waters^®^ 2414 RI detector (Waters Corp., Milford, MA) equipped with a Bio-Rad^®^ Aminex^®^ HPX-87H column conditioned at 65 °C using 5 mM sulfuric acid mobile phase at a flow rate of 0.6 mL/min for all separations. Cellulose conversions were calculated by:

$${\text{Cellulose}}\;{\text{conversion}}\;\left( {{\text{glucan}}\;{\text{yield}}\% } \right) = \frac{{\left( {{\text{Glucose}}\;\left( {\frac{{{\text{mg}}}}{{{\text{mL}}}}} \right) + \left( {{\text{Cellobiose}}\;\left( {\frac{{{\text{mg}}}}{{{\text{mL}}}}} \right) \times 1.053} \right)} \right) \times {\text{Reaction}}\;{\text{volume}}\;\left( {{\text{mL}}} \right) \times 0.9 \times 100}}{{{\text{Glucan}}\;{\text{in}}\;{\text{cellulosic}}\;{\text{substrate}}\;\left( {{\text{dry}}\;{\text{basis}}} \right)\;\left( {{\text{mg}}} \right)}}$$

where 0.9 accounts for the mass of water added to cellulose (glucan) during enzymatic hydrolysis and 1.053 accounts for the addition of water to form glucose from cellobiose.

### Average hydropathy of cellulases

GRAVY indices [54] of Cel7A, Cel6A, Cel7B and Cel5A were calculated through Sequence Manipulation Suite [62] using FASTA sequences from UniProtKB [63] Entry ID: A0A024RXP8, A0A024SH76, G0RKH9 and P07982, respectively.

## Additional files

**Additional file 1.** Additional experiments.

**Additional file 2.** Table for cellulose conversion data.

**Additional file 3.** Cellulase deactivation at the air–liquid interface.

## Abbreviations

FPU: filter paper units
CMC: critical micellar concentration
BSA: bovine serum albumin
Endo–Exo: endoglucanase–exoglucanase
GRAVY: grand average of hydropathy
