# Supplementary material for: Impacts of cellulase deactivation at the moving air–liquid interface on cellulose conversions at low enzyme loadings
Source: Biotechnol Biofuels. 2019 Apr 23;12:96. doi: 10.1186/s13068-019-1439-2 (PMC6477705; doi:10.1186/s13068-019-1439-2)
Supplement: Supplementary file 1 — Additional file 1. Additional experiments. [file 13068_2019_1439_MOESM1_ESM.pdf]

Additional File 1

Additional experiments

Impacts of Cellulase Deactivation at the Moving Air-Liquid Interface on Cellulose Conversions at Low Enzyme Loadings

Samarthya Bhagia, Charles E. Wyman, and Rajeev Kumar

Comparison of Accellerase® 1500 and Cellic® Ctec2 commercial cellulase preparations for effects of surfactant supplementation and shaking on Avicel cellulose conversions at a low enzyme loading

Our previous work used Dupont Accellerase® 1500, a commercial blend containing *T. reesei* enzyme with enhanced levels of β-glucosidase to cleave cellobiose into glucose. To be sure that the observed deactivation effects were not specific to this preparation, Avicel was hydrolyzed with Novozymes Cellic® CTec2, another commercial cellulase preparation." Fig. S1 shows that the maximum Avicel cellulose conversion at 1% glucan loading was 41% with 5 mg Cellic® CTec2 in shaken flasks, while 5 mg Tween supplementation in shaken flaks or keeping the flasks unshaken nearly doubled the maximum cellulose conversion. Unshaken flasks resulted in lower reaction rates than those from shaken flasks that were supplemented with surfactant. However, by comparison, the maximum Avicel cellulose conversions with Accellerase® 1500 for reactions without surfactant and shaking, with surfactant and shaking, and no surfactant and no shaking were 60%, 90%, and 94%, respectively (Fig. S1) [14]. These results show that deactivation of cellulase at the air-liquid interface was not specific to the Accellerase® 1500 preparation.

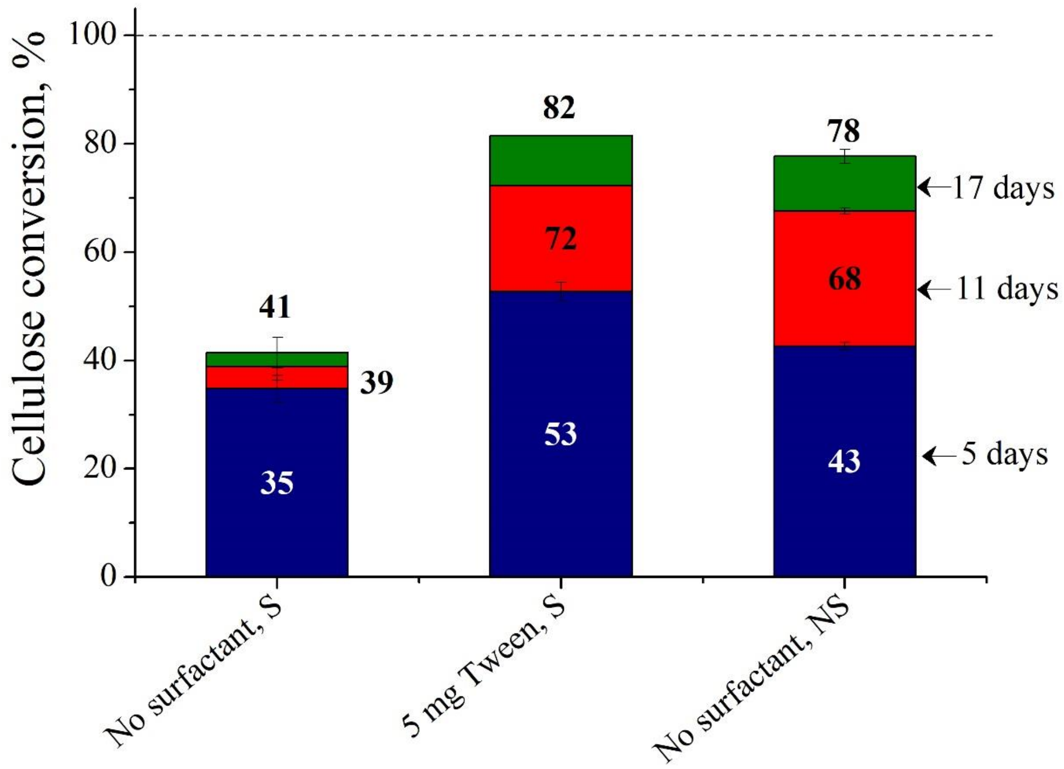

Fig. S1. Effects of surfactant supplementation and shaking on Avicel cellulose conversions using low loading of CTec2.

Cellulose conversions are reported after 5, 11, and 17 days of enzymatic hydrolysis of Avicel cellulose at 1% glucan loading using 5 mg cellulase protein (Novozymes® Cellic® CTec2) per g glucan in shaken flasks, 5 mg Tween 20 per g glucan-supplemented shaken flasks, and unshaken flasks, at 50 mL reaction volume in 125 mL Erlenmeyer flasks. S- shaking; NS- no shaking

Inactivation of cellulase due to shaking and "protection" from deactivation by BSA was observed in cellulases isolated from *Myrothecium verrucaria* [25, 27, 28], *Curvularia lunata*, *Aspergillus fumigatus*, *Chaetomium indicum*, *Penicillium rubrum*, and *Penicillium wortmanni* [26] since the early 1950s. Thus, deactivation of cellulase at the air-liquid interface is not limited to alterations in the glycosylation of cellulase secreted by hypercellulolytic mutant RUT-C30

isolated by screening for catabolite repression-resistant mutants of wild-type strain T. reesei QM6a in the 1970s. Thus, these results show that this deactivation phenomenon is inherent to cellulases but that preventing this deactivation can make conversions of nearly pure cellulose substrates exceptionally high at low enzyme loadings using such industrial preparations as Dupont's Accellerase® 1500 and Novozymes' Cellic® CTec2 if the reactions are carried out for a long enough time (Fig. S1).

Effects of changing interfacial area on Avicel cellulose conversions in unshaken flasks

It was previously shown that increasing the air-liquid interfacial area by roughly three and six times lowered Avicel cellulose conversion with 5 mg enzyme from 85% to 32% and 20%, respectively, in shaken flasks. And supplementation with 5 mg Tween reduced the drop in conversions [14]. Fig. S2 shows a similar setup but for unshaken flasks. In this case, enzymatic hydrolysis of Avicel glucan by 5 mg enzyme at a constant 10 mL reaction volume was carried out in 25, 125, and 500 mL flasks to achieve a gas-liquid interfacial area ratio of about 1: 3: 6. As a result of not shaking the flasks, high cellulose conversions were achieved regardless of flask size. In fact, 90% conversion was achieved in 500 mL flasks despite these having the largest interfacial area of reaction medium. This conversion is even better than the maximum of 76% achieved with 5 mg enzyme + 5 mg Tween in shaken 500 mL flasks in our previous work [14]. Unlike shaken flasks [14], Tween addition had little effect on cellulose conversions in any of the unshaken flasks.

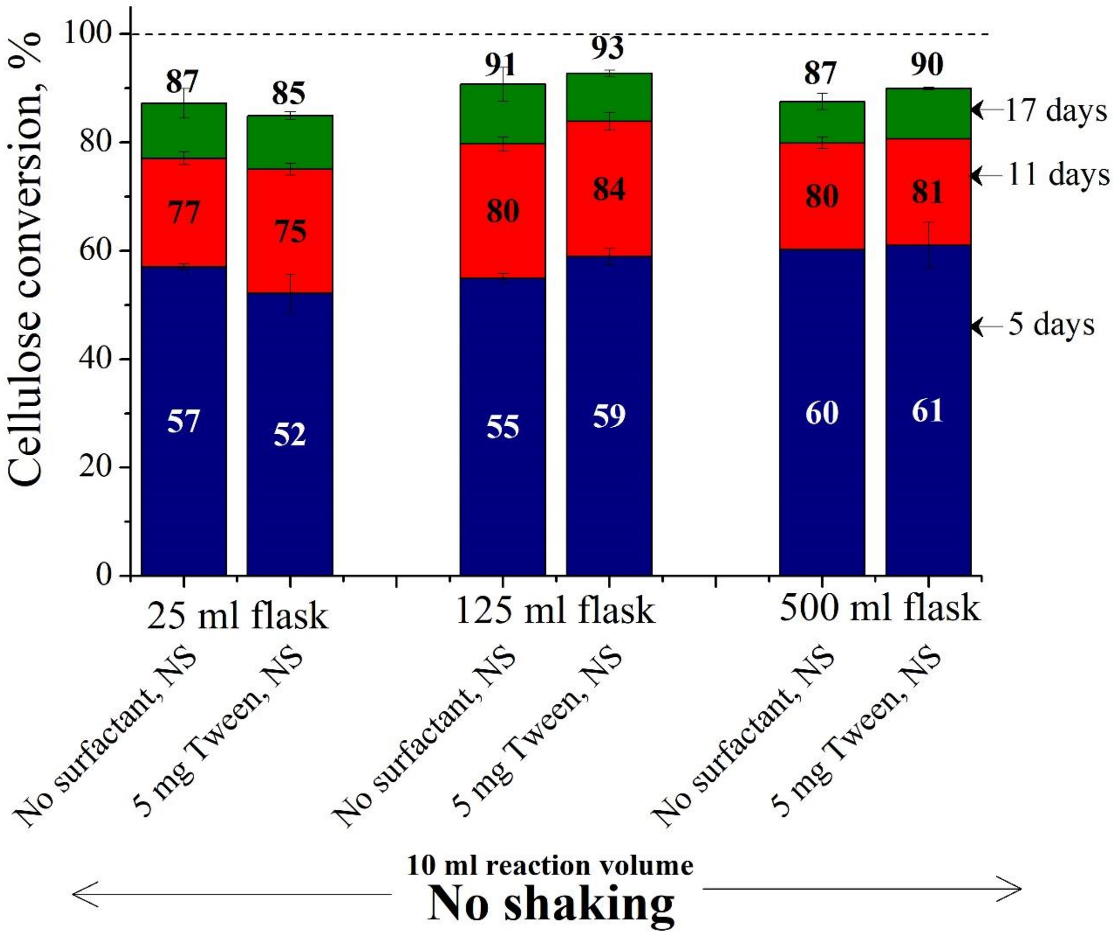

Fig. S2. Influence of interfacial area on Avicel cellulose conversions in unshaken flasks at low enzyme loading.
Cellulose conversions are reported after 5, 11, and 17 days of enzymatic hydrolysis of Avicel cellulose at 1% glucan loading using 5 mg cellulase protein (Accellerase® 1500) per g glucan in unshaken flasks and 5 mg Tween 20 per g glucan-supplemented unshaken flasks, at 10 mL reaction volume in 25, 125, and 500 mL Erlenmeyer flasks. NS- no shaking

Effects of surfactant supplementation and shaking on enzymatic conversions of beechwood xylan with xylanase

To evaluate whether the air-liquid interface has as dramatic an impact on xylanase as it did on cellulase, beechwood xylan was hydrolyzed with xylanase. Fig. S3 shows that there was no effect on conversion of beechwood xylan of either 5 mg Tween supplementation to 5 mg

xylanase (Accellerase XY) in shaken flasks or stopping shaking, as all flasks reached about 80% xylan conversion by 11 days. Unlike the lower reaction rates seen in cellulose-cellulase reactions at 1% glucan loading in unshaken flasks, reaction rates were the same in shaken and unshaken flasks in xylan-xylanase reactions at 1% xylan loading.

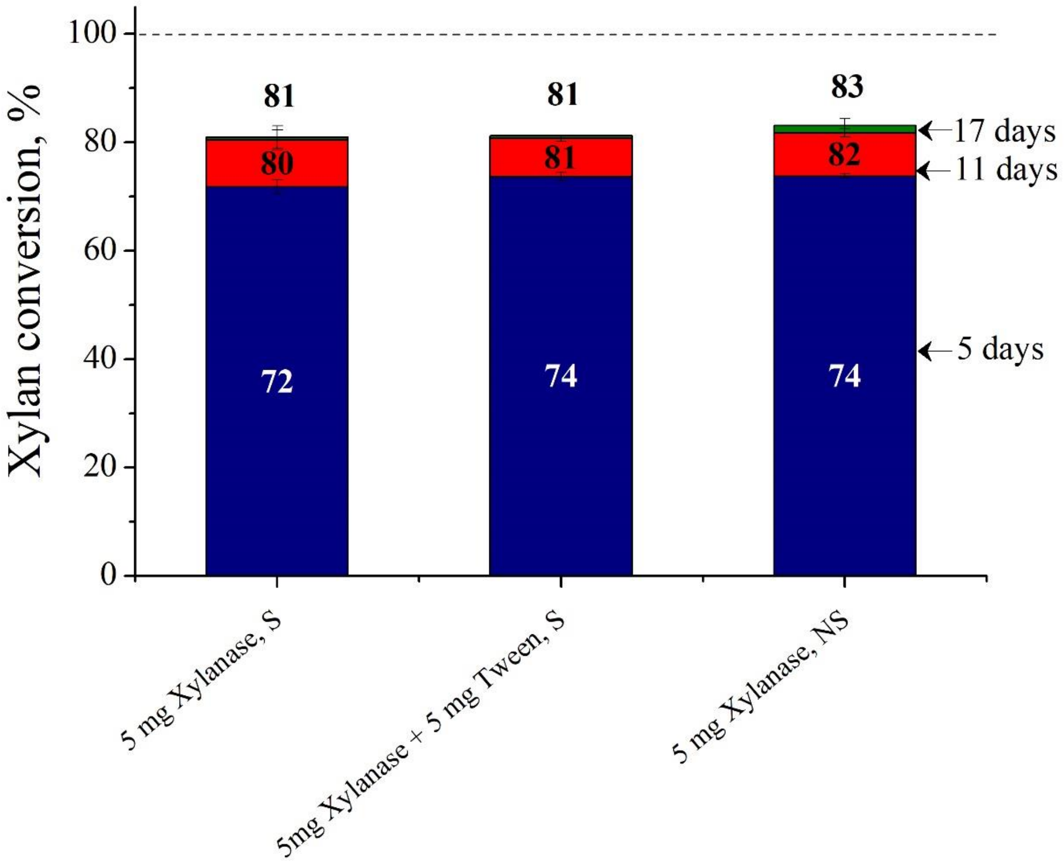

Fig. S3. Effects of surfactant supplementation and shaking on enzymatic conversions of beechwood xylan with xylanase**.**
Xylan conversions are reported after 5, 11, and 17 days of enzymatic hydrolysis of beechwood xylan at 1% xylan loading using 5 mg xylanase protein (Accellerase® XY) per g xylan and when supplemented with 5 mg Tween 20 per g xylan at 50 mL reaction volume in 125 mL Erlenmeyer shaken flasks. S- shaking; NS- no shaking

The small effect of shaking on beechwood xylan conversions showed that xylanase (Accellerase XY xylanolytic mixture) did not suffer from deactivation at air-liquid interface as much as cellulase at the same 5 mg per gram substrate loading for standard reaction conditions (Fig. S3). Xylan-xylanase reactions did not require shaking at the low substrate loading, as unlike crystalline cellulose, a portion of this xylan was soluble and the high reaction rates at 5 mg enzyme loading prevented high localized product concentrations that could have otherwise increased xylanase inhibition by xylose in unshaken flasks. Moreover, xylan hydrolysis solutions did not show any precipitates on vessel walls. Thus, xylanase loadings that are significantly lower than 5 mg protein per g xylan are required to see any negative effect of air-liquid interfacial deactivation of xylanase on isolated xylan conversions.
