# Supplementary material for: Impacts of cellulase deactivation at the moving air–liquid interface on cellulose conversions at low enzyme loadings
Source: Biotechnol Biofuels. 2019 Apr 23;12:96. doi: 10.1186/s13068-019-1439-2 (PMC6477705; doi:10.1186/s13068-019-1439-2)
Supplement: Supplementary file 2 — Additional file 2. Table for cellulose conversion data. [file 13068_2019_1439_MOESM2_ESM.pdf]

Substrate	Glucan loading %	Enz Loading (mg/g glucan)	Additive/condition	Vessel Type	Reaction Volume (ml)	Shaking (rpm)	Cellulose Conversions % After 5 days	After 11 days	After 17 days
**Fig. 1 Comparison of cellulosic substrates**									
Avicel	1	5	None	Erlenmeyer 125 ml	50	150	48	54	60
Avicel	1	5	5 mg Tween co-addition	Erlenmeyer 125 ml	50	150	71	85	89
Avicel	1	5	None	Erlenmeyer 125 ml	50	0	61	83	93
Cotton	1	5	None	Erlenmeyer 125 ml	50	150	30	39	41
Cotton	1	5	5 mg Tween co-addition	Erlenmeyer 125 ml	50	150	38	49	53
Cotton	1	5	None	Erlenmeyer 125 ml	50	0	29	43	50
Sigmacell	1	5	None	Erlenmeyer 125 ml	50	150	44	52	57
Sigmacell	1	5	5 mg Tween co-addition	Erlenmeyer 125 ml	50	150	57	71	75
Sigmacell	1	5	None	Erlenmeyer 125 ml	50	0	50	71	81
Alpha-Cellulose	1	5	None	Erlenmeyer 125 ml	50	150	49	58	62
Alpha-Cellulose	1	5	5 mg Tween co-addition	Erlenmeyer 125 ml	50	150	61	75	80
Alpha-Cellulose	1	5	None	Erlenmeyer 125 ml	50	0	50	74	82
Whatman paper	1	5	None	Erlenmeyer 125 ml	50	150	34	39	41
Whatman paper	1	5	5 mg Tween co-addition	Erlenmeyer 125 ml	50	150	44	56	59
Whatman paper	1	5	None	Erlenmeyer 125 ml	50	0	38	51	58
Whatman milled	1	5	None	Erlenmeyer 125 ml	50	150	62	74	80
Whatman milled	1	5	5 mg Tween co-addition	Erlenmeyer 125 ml	50	150	74	87	91
**Fig. 2 Effect of baffles in Erlenmeyer flasks**									
Avicel	1	5	None	Erlenmeyer 250 ml Baffle	50	150	12	13	13
Avicel	1	5	5 mg Tween co-addition	Erlenmeyer 250 ml Baffle	50	150	31	37	39
Avicel	1	5	None	Erlenmeyer 500 ml Baffle	50	150	9		
Avicel	1	5	5 mg Tween co-addition	Erlenmeyer 500 ml Baffle	50	150	32		
**Fig. 3 Cotton vs. Avicel at high enz loading**									
Avicel	1	30	None	Erlenmeyer 125 ml	50	150	97	100	

Avicel	1	30	100 mg Tween co-addition	Erlenmeyer 125 ml	50	150	101		
Avicel	1	30	None	Erlenmeyer 125 ml	50	0	82	96	99
Cotton	1	30	None	Erlenmeyer 125 ml	50	150	53	68	72
Cotton	1	30	100 mg Tween co-addition	Erlenmeyer 125 ml	50	150	57	70	74
Cotton	1	30	None	Erlenmeyer 125 ml	50	0	50	72	82
**Fig. 4 Shaking vs stirring**									
Avicel	1	5	None	Erlenmeyer 125 ml	50	150	48	54	60
Avicel	1	5	5 mg Tween co-addition	Erlenmeyer 125 ml	50	150	71	85	89
Avicel	1	30	None	Erlenmeyer 125 ml	50	150	97	100	
Avicel	1	30	100 mg Tween co-addition	Erlenmeyer 125 ml	50	150	101		
Avicel	1	5	None Stir	Erlenmeyer 125 ml	50	150	57	66	69
Avicel	1	5	5 mg Tween co-addition Stir	Erlenmeyer 125 ml	50	150	71	84	86
Avicel	1	30	None Stir	Erlenmeyer 125 ml	50	150	93	100	100
Avicel	1	30	100 mg Tween co-addition Stir	Erlenmeyer 125 ml	50	150	96	100	100
**Fig. 5 10 mg enz or 5 mg enz + 5 mg xyl**									
Avicel	1	10	None	Erlenmeyer 125 ml	50	150	78	87	91
Avicel	1	10	5 mg Tween co-addition	Erlenmeyer 125 ml	50	150	82	91	94
Avicel	1	10	None	Erlenmeyer 125 ml	50	0	66	89	96
Avicel	1	5	5 mg xyl	Erlenmeyer 125 ml	50	150	61	75	80
Avicel	1	5	5 mg xyl+5mg tween	Erlenmeyer 125 ml	50	150	66	79	84
Avicel	1	5	5 Xyl	Erlenmeyer 125 ml	50	0	56	80	88
**Fig. 6 Filling of reactor and tubes**									
Avicel	1	5	Fill reactor 146 ml reac. vol.	Erlenmeyer 125 ml	50	150	20	35	50
Avicel	1	5	Fill reactor 146 ml reac. vol. + 5 Tween	Erlenmeyer 125 ml	50	150	19	34	51
Avicel	1	5	140 ml Horizontal	Long	140	150	53	74	80
Avicel	1	5	140 ml Horizontal + 5 mg Tween	Long	140	150	54	73	79
Avicel	1	5	10 ml Horizontal	Long	10	150	43	53	56
Avicel	1	5	10 ml Horizontal + 5 mg Tween	Long	10	150	61	78	82
**Fig. 7 High solids loading**									
Avicel	15	5	No	Erlenmeyer 125 ml	50	150	25	30	32
Avicel	15	5	5 mg Tween co-addition	Erlenmeyer 125 ml	50	150	57	59	66
Avicel	15	5	None	Erlenmeyer 125 ml	50	0			61
Avicel	15	5	5 mg Tween co-addition	Erlenmeyer 125 ml	50	0			62

**Fig. 8 Increasing Tween 20 concentration**									
Avicel	1	5	5 mg Tween co-addition	Erlenmeyer 125 ml	50	150	71	85	89
Avicel	1	5	100 mg Tween co-addition	Erlenmeyer 125 ml	50	150	66	81	86
Avicel	1	5	500 mg Tween co-addition	Erlenmeyer 125 ml	50	150	64	0	75
**Fig. S1 Comparison of Accellerase 1500 vs. Cellic Ctec 2**									
Avicel	1	5	None	Erlenmeyer 125 ml	50	150	48	54	60
Avicel	1	5	5 mg Tween co-addition	Erlenmeyer 125 ml	50	150	71	85	89
Avicel	1	5	None	Erlenmeyer 125 ml	50	0	61	83	93
Avicel	1	5	Acc1500 Replaced w/ CTec2	Erlenmeyer 125 ml	50	150	35	39	41
Avicel	1	5	Acc1500 Replaced w/ CTec2 + 5 Tween co-addition	Erlenmeyer 125 ml	50	150	53	72	82
Avicel	1	5	CTec2	Erlenmeyer 125 ml	50	0	43	68	78
**Fig S2. Changing interfacial area in unshaken flasks**									
Avicel	1	5	None	Erlenmeyer 25 ml	10	0	57	77	87
Avicel	1	5	5 mg Tween co-addition	Erlenmeyer 25 ml	10	0	52	75	85
Avicel	1	5	None	Erlenmeyer 125 ml	10	0	55	80	91
Avicel	1	5	5 mg Tween co-addition	Erlenmeyer 125 ml	10	0	59	84	93
Avicel	1	5	None	Erlenmeyer 500 ml	10	0	60	80	87
Avicel	1	5	5 mg Tween co-addition	Erlenmeyer 500 ml	10	0	61	81	90
**Fig. S3 Isolated xylan hydrolysis with xylanase**									
Xylan	1	5	Xylanase Only	Erlenmeyer 125 ml	50	150	72	80	81
Xylan	1	5	Xylanase Only + 5 mg Tween co-addition	Erlenmeyer 125 ml	50	150	74	81	81
Xylan	1	5	None	Erlenmeyer 125 ml	50	0	74	82	83
